# Interactions between flagellar and type III secretion proteins in *Chlamydia pneumoniae*

**DOI:** 10.1186/1471-2180-10-18

**Published:** 2010-01-22

**Authors:** Chris B Stone, David C Bulir, Jodi D Gilchrist, Raman K Toor, James B Mahony

**Affiliations:** 1 M.G. DeGroote Institute for Infectious Disease Research, Faculty of Health Sciences and the Department of Pathology and Molecular Medicine, McMaster University, and the Father Sean O'Sullivan Research Centre, St. Joseph's Healthcare, Hamilton, Ontario, Canada

## Abstract

**Background:**

Flagellar secretion systems are utilized by a wide variety of bacteria to construct the flagellum, a conserved apparatus that allows for migration towards non-hostile, nutrient rich environments. *Chlamydia pneumoniae *is an obligate, intracellular pathogen whose genome contains at least three orthologs of flagellar proteins, namely FliI, FlhA and FliF, but the role of these proteins remains unknown.

**Results:**

Full length FliI, and fragments of FlhA, FliF, and FliI, were cloned and expressed as either GST or His tagged proteins in *E. coli*. The GST-tagged full length FliI protein was shown to possess ATPase activity, hydrolyzing ATP at a rate of 0.15 ± .02 μmol min^-1 ^mg^-1 ^in a time- and dose-dependant manner. Using bacterial-2-hybrid and GST pull-down assays, the N-terminal domain of FliI was shown to interact with the cytoplasmic domain of FlhA, but not with FliF, and the cytoplasmic domain of FlhA was shown to interact with the C-terminus of FliF. The absence of other flagellar orthologs led us to explore cross-reaction of flagellar proteins with type III secretion proteins, and we found that FliI interacted with CdsL and CopN, while FlhA interacted with CdsL and Cpn0322 (YscU ortholog CdsU).

**Conclusions:**

The specific interaction of the four orthologous flagellar proteins in *C. pneumoniae *suggests that they interact *in vivo *and, taken together with their conservation across members of the chlamydiae *sps*., and their interaction with T3S components, suggests a role in bacterial replication and/or intracellular survival.

## Background

*Chlamydia pneumoniae *is a gram negative, obligate intracellular pathogen that has been associated with community-acquired pneumonia [1], atherosclerosis [2], arthritis [3], and Alzheimer's disease [4]. *C. pneumoniae *is characterized by a unique, biphasic life cycle beginning with an infectious, metabolically attenuated elementary body (EB). Chlamydial invasion is initiated by attachment of the EB to the host eukaryotic cell membrane and recruitment of actin to the site of attachment. This remodeling of the actin cytoskeleton is thought to be mediated by the type III secretion (T3S) effector protein, the translocated actin recruitment protein (TARP), which facilitates chlamydial entry into the host cell [5,6]. Bacterial uptake involves modulation of the host MEK-ERK pathway and PI 3-kinase, possibly through the action of T3S effectors [7,8]. Once internalized, the remainder of the chlamydial life cycle takes place within a parasitophorous membrane-bound vesicle known as an inclusion, where EBs differentiate into the non-infectious, metabolically active form, termed a reticulate body (RB). Within the inclusion, RBs acquire amino acids, nucleotides, lipids and cholesterol from the host cell, events possibly orchestrated via T3S across the inclusion membrane, while at the same time inhibiting apoptosis to ensure survival [9-11]. Golgi fragmentation appears to be a crucial step in intercepting host pathways to obtain these nutrients and compounds, as well as in the maturation of the chlamydiae *sps. *within the inclusion [12]. The RB interacts with the inclusion membrane until such time as inclusion membrane RB docking sites are no longer available and an unknown signal triggers detachment of the RB from the inclusion membrane followed by asynchronous differentiation into EBs [13,14]. The newly formed EBs then exit the cell by either cellular lysis or a packaged release mechanism termed extrusion [15].

*C. pneumoniae *encodes a full set of T3S genes scattered throughout the genome in several fragmented operons. The T3S injectisome has a high amount of paralogy to the flagellar secretion system in structure and in function. In the T3SS, CdsN is the ATPase that aids in shuttling effectors through the needle, and is paralogous to FliI [16]. CdsL is orthologous to YscL and paralogous to FliH. In *Yersinia*, YscL is the ATPase tethering protein and functions to down-regulate enzymatic activity of YscN [17]. CopN, orthologous to YopN, is believed to function as a regulator of the system which plugs the injectisome pore prior to activation of T3S and is a known effector protein [18]. CdsU, orthologous to YscU, plays an important role is substrate specificity and substrate switching from structural components to effector proteins upon host cell contact [19]. Recently, several reports have emerged characterizing protein interactions within the *C. pneumoniae *T3SS, describing novel protein complexes that form at the inner membrane. Johnson et al have shown that CdsD, a unique protein orthologous to YscD that contains two fork-head associated domains, interacts with the predicted *C. pneumoniae *ATPase tethering protein, CdsL, and CdsQ, a cytosolic component of the inner membrane that presumably forms the bulk of the T3S C-ring [20]. Stone et al extended these findings to show that CdsN, the ATPase, is also involved in this complex as well as interacting with the proposed plug protein, CopN [16].

Flagellar motility is an ancient, conserved mechanism that may have evolved from the same ancestor as T3S [21]. This motility facilitates bacterial migration towards less hostile environments. In non-motile bacteria, however, the presence of flagella would be evolutionarily redundant and energetically expensive, unless the proteins played a role in another mechanism involving bacterial replication or survival. Although *C. pneumoniae *is thought to be a non-motile bacteria, it has been shown to contain at least three orthologs of flagellar genes, namely *flhA, fliF, and fliI *[22,23]. Microarray and proteomic experiments have suggested that these genes are expressed at mid-cycle [23]. The proteins encoded by these genes are paralogs of the T3S proteins CdsV, CdsJ and CdsN, respectively. In motile bacteria, FlhA orthologs are integral membrane proteins required for flagellin export and swarming differentiation which interact with soluble components of the flagellar system [24,25]. FliF orthologs are integral membrane components that form the membrane and supramembrane (MS) ring [26]. FliF forms a base for the other membrane components to form a molecular pore, through which components of the flagella that exist outside the cell membrane are exported. The flagellar ATPase, FliI orthologs, provide energy for construction of the flagellum by aiding in export of flagellar proteins outside the bacterial cell where the proteins form molecular complexes [27,28]. The presence of FliI, FlhA and FliF in *C. pneumoniae *is not sufficient to form a fully functional flagellar apparatus but they could potentially form a rudimentary base for a flagellum structure [29]. In chlamydiae, the identity of other proteins (if they exist) that play important roles in the flagellar apparatus is currently pending, but it is possible that the flagellar apparatus, if it exists, is a hybrid structure of *C. pneumoniae *T3S and flagellar proteins. Another possibility is that flagellar proteins are involved in T3S, aiding in secretion of effector proteins or structural components. In *Pseudomonas*, there is evidence to support that flagellar assembly actually antagonizes the T3SS, suggesting a negative cross-regulation of the two systems [30]. No interaction between chlamydial T3S and flagellar components, however, has been reported to our knowledge.

The protein interactions that occur within the bacterial flagellar system have been characterized previously [29,31,32]. Genetic evidence, followed by direct biochemical assays, suggests an interaction of FlhA and FliF [33,34]. The C-terminal end of FlhA, which is predicted to be cytoplasmic, is known to interact with the soluble components of the flagellar system such as FliI, FliH and FliJ [34,35]. FliH acts as a negative regulator of the flagellar ATPase, FliI, and binds FliI as a homodimer, forming a trimeric (FliI)(FliH)_2 _complex [36-38]. FliJ, a second soluble component which interacts with FlhA, acts as a general chaperone for the flagellar system to prevent premature aggregation of export substrates in the cytoplasm, and also interacts with the FliH/FliI complex [39]. This complex of FliI/FliH/FliJ is believed to be crucial for selection of export substrates and construction of the flagellar apparatus, although the proton motive force could play a role in the actual secretion of flagellar proteins [28,40]. In *C. pneumoniae*, FliH and FliJ have not been annotated in the genome.

FliI, the putative *C. pneumoniae *flagellar ATPase ortholog, has significant amino acid similarity with both CdsN, the *C. pneumoniae *T3S ATPase, and FliI, the *Salmonella *flagellar ATPase, suggesting that it possesses enzymatic activity. Here we report an initial characterization of FliI, the flagellar ATPase, and show that it hydrolyzes ATP at a rate similar to that of its T3S ATPase paralog CdsN as well as orthologs in other bacteria [16,41,42]. We have also characterized the protein-interactions occurring between FliI, FliF and FlhA, demonstrating a direct interaction of FliI and FlhA, and FlhA and FliF. As well as interactions between the flagellar proteins, we have also characterized four novel interactions between the flagellar and T3S components. The role of these interactions in the chlamydial replication cycle is discussed.

## Results

### Sequence analysis of FliI, FlhA and FliF

**Figure 1:**
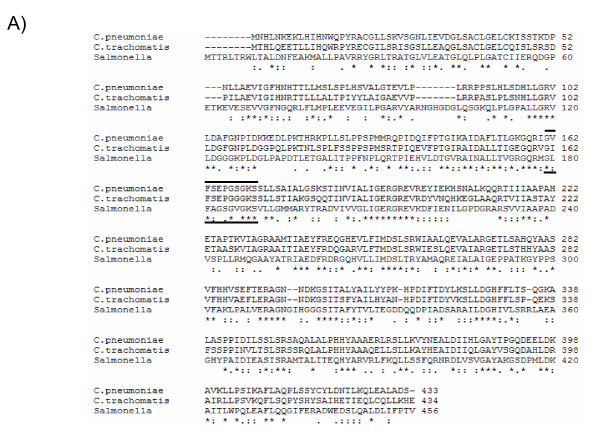
**Sequence conservation of FliI from *C. pneumoniae *with *C. trachomatis *and *Salmonella***. Sequence alignment (ClustalW) of the full length FliI protein from *C. pneumoniae*, *C. trachomatis*, and *Salmonella*. Asterisk refers to identical amino acids, a double dot refers to a conserved substitution and a single dot refers to a semi-conserved substitution. Outlined is the conserved P loop region in the Walker A domain.

FliI (Cpn0858) is 434 amino acids in length with a predicted molecular mass of 47.5 kDa and a pI of 8.00. FliI has significant sequence similarity with the β subunit of the F_0_F_1_-ATPase and both its type III secretion paralog, CdsN, and other flagellar ATPases, although the sequence similarity is most striking in the ATPase domain [43]. FliI has a non-conserved N-terminal region (amino acids 1-150) which may be important for mediating protein-protein interactions, a catalytic domain (amino acids 150-329), containing a conserved P loop, Walker A and B domains, and a C-terminal non-conserved domain (amino acids 329-434) of unknown function. FliI has a 34 percent sequence similarity to CdsN, the *C. pneumoniae *T3S ATPase, and 36 percent similarity with FliI from *Salmonella*. The active domain of FliI has the most similarity to its paralogs and orthologs, while the N- and C-terminal regions have the lowest amount of similarity (Figure 1).

FlhA (Cpn0363) is 583 amino acids in length with a predicted molecular mass of 65.6 kDa and a pI of 5.60. The FlhA paralog in *C. pneumoniae *is the T3S protein CdsV. FlhA has seven predicted transmembrane regions in the N-terminal half of the protein (FlhA_1-308_), while the C-terminal half of the protein is predicted to be cytoplasmic (TMpred). FlhA from *C. pneumoniae *has 21 percent sequence orthology with FlhA from *Salmonella*.

FliF (Cpn0860) is 342 amino acids in length with a predicted molecular mass of 38.2 kDa and a pI of 9.5. The FliF paralog in *C. pneumoniae *is the T3S protein CdsJ. FliF has two predicted TM regions, one located near the N-terminus and one located near the C-terminus. FliF from *C. pneumoniae *is only 15 percent similar to FliF from *Salmonella.*

### Expression and ATPase activity of FliI

**Figure 2:**
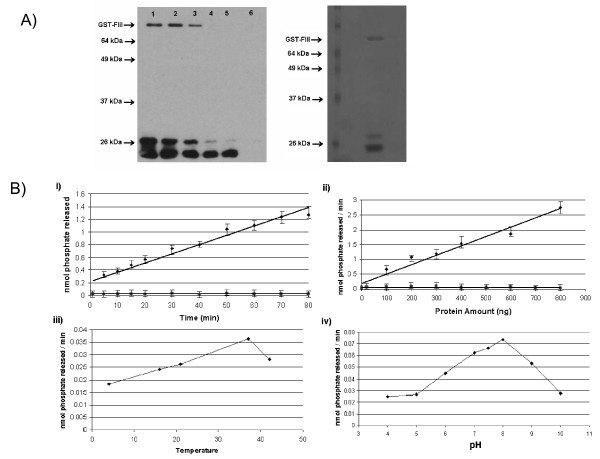
**Expression, purification, and optimal conditions for the time- and dose-dependant ATPase FliI**. **A**: GST-FliI eluted off a glutathione column (1 mL aliquots) and electrophoresed on an 11% SDS-PAGE gel and either probed for by anti-GST Western blot (left) or stained by Coomassie blue (right). GST-FliI migrated at approximately 73 kDa, its predicated molecular mass. Numbers refer to the eluted fraction. **B**: i) Time course of purified GST-FliI ATP hydrolysis (diamonds) and GST-CopN ATP hydrolysis as a negative control (squares). ii) Inorganic phosphate released at different concentrations of GST-FliI (diamonds) and GST-CopN as a negative control (squares) iii) GST-FliI ATPase activity at either 4°C, 16°C, 23°C, 37°C or 42°C. iv) GST-FliI ATPase activity at varying pH.

FliI has significant similarity with many characterized ATPases, and this led us to explore the ATPase activity of this protein. Purified GST tagged FliI was tested for its ability to hydrolyze ATP using the malachite green binding assay. Figure 2A shows that GST-FliI was essentially free of contaminating proteins by SDS-PAGE and anti-GST Western blot (left) or Coomassie blue stain (right). GST-FliI hydrolyzed ATP in a dose- and time-dependant manner at a rate of 0.15 ± .02 μmol min^-1 ^mg^-1 ^(Figure 2B i and ii, diamonds). This level of activity is comparable to other flagellar ATPases as well as the T3S paralog, CdsN [16,41,42]. ATPase activity of GST-FliI peaked at 37°C, and at a pH of 8.0 (Figure 2 iii and iv). Another GST-tagged protein, GST-CopN, purified in the same manner as GST-FliI had negligible ATPase activity (Figure 2B i and ii, squares).

### FlhA interacts with FliF

**Figure 3:**
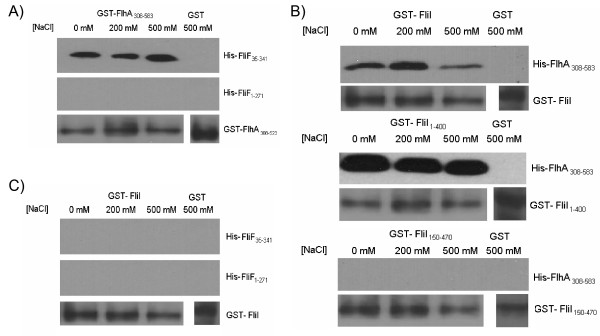
**Interaction between the flagellar components using GST pull-down assays**. **A**: GST- FlhA_308-583 _was bound to glutathione beads and was used to pull down either His-FliF_35-341 _or His-FliF_1-271 _from an *E. coli *lysate. Beads were harvested by centrifugation and washed with either 0 mM, 200 mM or 500 mM NaCl and probed for His-tagged protein by Western blot using anti-his antibody. GST- FlhA_308-583 _co-purified with His-FliF_35-341 _but not His-FliF_1-271 _while GST alone did not co-purify with either. GST-FlhA_308-583 _is shown as a loading control. **B**: Full length GST-FliI, GST-FliI_1-400_, or GST-FliI_150-470 _were bound to glutathione beads and were used to pull down His-FlhA_308-583 _from an *E. coli *lysate. Full length GST-FliI and GST-FliI_1-400 _were able to co-purify with FlhA_308-583 _while GST-FliI_150-470 _was not. GST alone was not able to co-purify with His-FlhA_305-583_. **C**: Full length GST-FliI was bound to glutathione beads and used to pull down His-FliF_35-341 _and His-FliF_1-271_. GST-FliI did not co-purify with either FliF fragment.

FlhA is known to interact with the MS ring protein, FliF, in other flagellar systems [33,34]. We explored the interactions of these two proteins in *C. pneumoniae*. Two fragments of FliF were cloned and expressed as His-tagged proteins. His-FliF_1-271 _lacked the distal C-terminal 70 amino acids while His-FliF_35-341 _lacked the N-terminal 35 amino acids. Each fragment contained only one of the two predicted TM regions. FliF_1-271 _migrated with an apparent molecular weight of 30 kDa, while His-FliF_35-341 _migrated at 34 kDa. FlhA was cloned and expressed as a soluble fragment with either a GST or His tag. FlhA_308-583 _encoded the C-terminal half of the protein, lacking the stretch of seven TM domains. Expression and detection of His-FlhA_308-583 _used as the bait protein in GST pull-down assays migrated at the expected molecular weight of 30 kDa. We used the bacterial-2-hybrid assay to test for interactions between FliF and FlhA. Full length FlhA interacted significantly with full length FliF, with a β-galactosidase activity of 847.2 ± 21.2 units of activity, as compared with a negative control value of 412.0 ± 82.4 units of activity (Table 1). We next used GST pull-downs to confirm the interactions found by the bacterial-2-hybrid system and to determine the exact regions of the proteins mediating these interactions (Figure 3A). All protein complexes were washed with either low or high salt buffers containing 0.1% Triton X-100 to dissociate spurious protein-protein interactions. GST-FlhA_308-583 _co-purified with His-FliF_35-341 _but not His-FliF_1-271_, suggesting that the C-terminus of FliF (amino acids 271-341) is required for interactions with the cytoplasmic portion of FlhA.

**Table 1:** Interaction between the flagellar proteins of *C. pneumoniae *using the Bacterial-2-hybrid System

Plasmids	β-Galactosidase Activity in units/mg bacteria	Protein Functions
**Negative Control**		
pT18 + pT25	412.0 ± 82.4	pT18: Empty vector
**Positive Control**:		pT25: Empty vector
pT18-PknD + pT25-CdsD-FHA-2	996.3 ± 50.0	FliI: Putative flagellar ATPase
**Negative Interactions**:		FliF: Putative flagellar MS ring protein
pT18-FliI + pT25-FliF	396.4 ± 32.1	FlhA: Putative flagellar integral membrane
pT18-FliF + pT25-Cpn0859	421.1 ± 25.9	protein
pT18-FliI + pT25-Cpn0706	404.4 ± 19.5	Cpn0859: Hypothetical *C. pneumoniae*
pT18-Cpn0706 + pT25-FlhA	443.0 ± 32.3	protein
**Positive Interactions**:		Cpn0706: Putative T3S chaperone
pT18-FliF + pT25-FlhA	847.2 ± 21.2	CdsL: Putative T3S ATPase tethering
pT18-FliI + pT25-FlhA	942.9 ± 123.1	protein
pT18-FliI + pT25-CdsL	874.3 ± 59.3	CopN: Putative T3S plug protein
pT18-FliI + pT25-CopN	943.2 ± 74.2	Cpn0322: Putative CdsU ortholog
pT18-Cpn0322 + pT25-FlhA	779.9 ± 32.7	
pT18-CdsL + pT25-FlhA	832.1 ± 23.3	

* FliI, FliF, FlhA, CdsL, CopN, Cpn0706 and Cpn0322 were cloned into both the pT18 and pT25 vectors. The bacterial-2-hybrid was performed in triplicate as described in the Materials and Methods section. Empty pT18 and pT25 vectors were used as a negative control while pT18-PknD + pT25-CdsD-FHA-2 was used as a positive control. The cut off for a positive interaction (577 units of activity/mg protein), is the mean of the negative control values (empty pT18 + pT25) plus two standard deviations obtained from 20 assays.

### FliI interacts with FlhA

In orthologous systems, it has been shown that FlhA interacts with several soluble

components of the flagellar machinery, including the ATPase, FliI [34]. Therefore, we investigated the possibility of whether FlhA interacts with FliI in *C. pneumoniae*. The bacterial-2-hybrid system was initially used to screen for potential protein interactions. FlhA interacted with FliI, with β-galactosidase activity of 942.9 ± 123.1 units of activity as compared to the negative control with a value of 412.0 ± 82.4 units of activity (Table 1). To confirm these protein-protein interactions we used GST pull-down assays (Figure 3B). Initially FliI was cloned as three constructs, full length FliI, a C-terminal truncation of FliI (FliI_1-400_) and a N-terminal truncation of FliI (FliI_150-471_). These three constructs were tested for interaction with the His-FlhA_308-583 _construct. Full length GST-FliI co-purified with His-FlhA_308-583_, suggesting that the cytoplasmic fragment of FlhA contains the interactive domain. To determine the region of FliI that interacts with FlhA, we reacted the two truncation constructs of FliI with the cytoplasmic domain of FlhA. Only FliI_1-400 _was able to co-purify with FlhA, and not FliI_150-471_, suggesting that the FlhA binding domain resides in the N-terminal 150 amino acids of FliI (Figure 3B). We next wanted to know if FliI interacts with FliF. We therefore reacted GST-FliI against the two FliF constructs and found that there was no co-purification, suggesting that any interaction between FliI and FliF, if there is an association, would seem to be indirect and mediated through the action of FlhA or other intermediate proteins (Figure 3C).

### Cpn0859 interacts with FliI and FlhA

**Figure 4:**
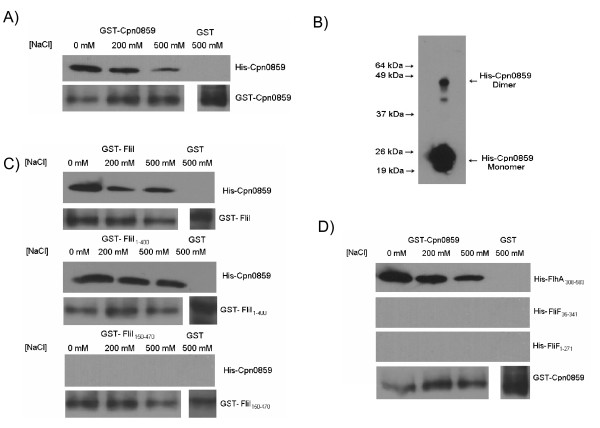
**Interaction of His-CPn0859 and GST-Cpn0859, and dimerization of His-Cpn0859**. **A**: Full length GST-Cpn0859 was bound to glutathione beads and was used to pull down full length His-Cpn0859 from an *E. coli *lysate, as seen in Figure 3. GST-Cpn0859 co-purified with His-Cpn0859. GST alone did no co-purify with His-Cpn0859, and GST-Cpn0859 is shown as a loading control. **B**: Full length His-Cpn0859 was fixed with formaldehyde for 10 minutes prior to being electrophoresed on an 11% PAGE gel and probed for by anti-His Western blot. Cpn0859 monomers can be seen migrating at approximately 22 kDa while the formation of a dimer can be seen migrating at approximately 44 kDa. **C**: Full length GST-FliI, GST-FliI_1-400_, or GST-FliI_150-470 _were bound to glutathione beads and were used to pull down His-Cpn0859 from an *E. coli *lysate. They were washed in the same manner as above, and only full length GST-FliI and GST-FliI_1-400 _were able to co-purify with His-Cpn0859. **D**: Full length GST-Cpn0859 was bound to glutathione beads and was used to pull down either His-FlhA_308-583_, His-FliF_35-341 _or His-FliF_1-271 _from an *E. coli *lysate. GST-Cpn0859 co-purified with His- FlhA_308-583, _but not His-FliF_35-341 _or His-FliF_1-271 _while GST alone did no co-purify with either.

*Cpn0859 *is a predicted 179 amino acid protein with a PI of 6.10 and a molecular mass of 20.3 kDa. The *Cpn0859 *ORF is encoded directly upstream of *fliF *and downstream of *fliI*, the flagellar ATPase. Based on its location relative to FliI and FliF, we hypothesized that it may interact with other flagellar components. We used GST pull-down assays to explore this possibility. Initial GST pull-downs indicated that full length His-Cpn0859 interacts with GST-Cpn0859, suggesting the presence of a dimerization domain (Figure 4A). To explore this observation we treated Cpn0859 with formaldehyde prior to PAGE and observed the presence of a monomer and a dimer, migrating with apparent molecular weights of 22 kDa and 45 kDa (Figure 4B). We next explored the possible interaction of Cpn0859 with other flagellar proteins in *C. pneumoniae*. Using GST pull-downs, His-Cpn0859 co-purified with the full length GST-FliI protein as well as the GST-FliI_1-400 _protein, but not GST-FliI_150-471 _(Figure 4C). This suggests that Cpn0859 binds to the N-terminus of FliI. GST pull-down assays showed an interaction between Cpn0859 and the FlhA_308-583 _protein, the cytoplasmic domain of FlhA (Figure 4D). Cpn0859 did not co-purify with either FliF_35-341 _or FliF_1-271 _(Figure 4D), suggesting that Cpn0859 does not interact with FliF.

### FliI and FlhA interact with T3S components

**Figure 5:**
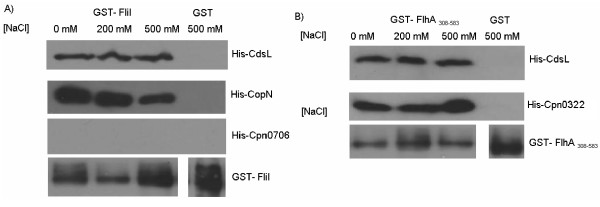
**Interaction of FliI and FlhA with T3S components**. **A**: Full length GST-FliI was bound to glutathione beads and were used to pull down His-CdsL, His-CopN and His-Cpn0706. GST-FliI co-purified with both His-CdsL and His-CopN, but not His-Cpn0706. GST alone was not able to co-purify with any of the proteins. GST-FliI is shown as a loading control. **B**: GST-FlhA_308-583 _was bound to glutathione beads and used to pull down His-CdsL and His-Cpn0322. GST-FlhA_308-583 _co-purified with both CdsL and Cpn0322. GST alone did not co-purify with either protein. GST-FlhA_308-583 _is shown as a loading control. C:

Since *Chlamydia *have no apparent flagella, we investigated whether the flagellar proteins FliI, FlhA and FliF interact with T3S components. Using bacterial-2-hybrid screening we found that FliI and FlhA interacted with CdsL, the putative T3S ATPase regulator and tethering protein, with a β-galactosidase activity of 874.3 ± 59.3 and 832 ± 23.3 units of activity, respectively. FliI also interacted with CopN, the putative T3S plug protein, with a β-galactosidase activity of 943.2 ± 74.2 units of activity. We also found that FlhA interacted with the putative YscU ortholog, CdsU, with a β-galactosidase activity of 779.9 ± 32.7 units of activity, as well as CdsL, with a β-galactosidase activity of 832.1 ± 23.3 units of activity (Table 1). To corroborate these findings we utilized GST pull-down assays and showed that GST-FliI interacted with CdsL and CopN, but not Cpn0706 (Figure 5A), and GST-FlhA co-purified with both CdsL and CdsU (Figure 5B). Control GST coated beads did not co-purify with CdsL, CopN or CdsU.

## Discussion

Sequencing of several Chlamydial genomes revealed a conserved set of flagellar orthologs, despite the fact that *C. pneumoniae *lack a flagellum and are considered non-motile bacteria [22,23]. Here we report an initial characterization of three annotated flagellar proteins of *C. pneumoniae*, FliI, FlhA and FliF, demonstrating ATPase activity of FliI and interactions between these flagellar orthologs. We have demonstrated that FliI hydrolyzes ATP in a linear, time-and dose-dependant manner, with optimal activity at a pH of 8.0 and a temperature of 37°C. FliI also interacts with the cytoplasmic domain of FlhA, while FlhA interacts with the C-terminal region of the FliF protein. No direct interaction of FliI and FliF was detected. Also, we have characterized an interaction of both FlhA and FliI with Cpn0859, a fourth unannotated protein. We also show that FliI interacts with CdsL and CopN, two T3S components, while FlhA interacts with CdsL and a third T3S component, CdsU. Collectively, this data suggests that the flagellar proteins of *C. pneumoniae *interact in a specific way with T3S proteins and may play an important, as yet unidentified role in the chlamydial replication cycle.

FliI hydrolyzes ATP in a linear, time- and dose-dependant manner at a rate of 0.15 ± .02 μmol min^-1 ^mg^-1^. This rate is typical of other secretion ATPases such as CdsN, EscN, or FliI from other bacterial species [16,41,42]. The optimal pH for FliI ATPase activity is 8.0, which is the same as that for other flagellar ATPases [42]. Extreme low or high pH greatly reduced the activity, possibly due to protein denaturation. Also, the enzyme activity peaked at a temperature of 37°C and declined substantially beyond that. Although the formation of higher-order complexes was not explored here, other flagellar ATPases are thought to form a hexameric complex [44].

The presence of three flagellar genes in *chlamydiae *is intriguing since *chlamydiae *are thought to be non-motile and not to possess flagella. FliF, FlhA and FliI alone do not contain all the necessary components for a functional flagella or secretion apparatus, however, a rudimentary basal body or pore complex could be formed by these three components. It is known that the most rudimentary flagellar structure that can be assembled is the MS ring, which consists of only the FliF protein [29]. We have shown that these proteins interact with one another (FliI, FlhA and FliF), most likely at the inner membrane of *C. pneumoniae*. The interaction between FliI and FlhA is mediated by the N-terminal 150 amino acids of FliI and appears to be specific since it is not disrupted by high salt (500 mM). Only the cytoplasmic domain of FlhA (amino acids 308-583) was utilized in the GST pull-down, suggesting that any protein interactions that occur are within this region. Protein interaction studies with the full length FlhA protein are difficult due to the presence of seven transmembrane domains rendering full length FlhA insoluble and making this portion of the protein unable to bind to soluble flagellar components. Since FlhA is known to interact with soluble components of the flagellar apparatus in other bacteria, it is expected that the cytoplasmic domain mediates an interaction with FliI [25]. FliF is known to form the MS ring in flagellated bacteria, and is one of the first components of the flagellar basal body to be incorporated into the membrane [26,29]. We detected an interaction of the C-terminal 70 amino acids of FliF with the cytoplasmic domain of FlhA. These interactions were also stable in 500 mM NaCl, suggesting that the interaction is specific. We did not, however, detect any interaction between FliI and FliF, suggesting that any interaction between those two components may be mediated through the action of another protein, possibly FlhA

**Figure 6:**
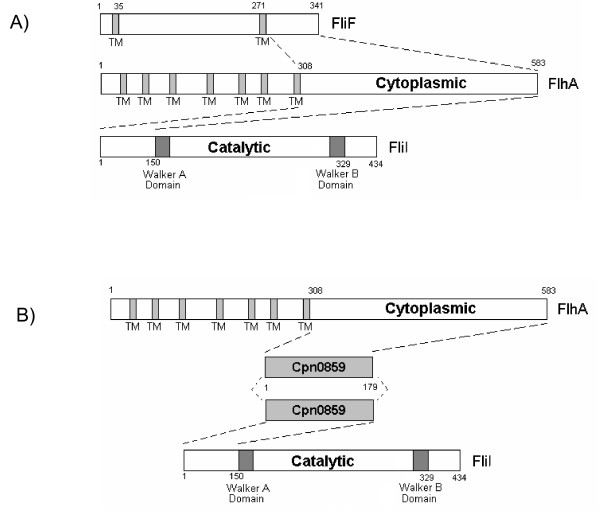
**Interacting regions between FliI and FlhA, FliF, and Cpn0859**. **A**: FliF contains two transmembrane regions and interacts with the cytosolic domain of FlhA by its extreme C-terminal end. FlhA contains seven transmembrane regions and interacts with the N-terminal region of FliI by its cytoplasmic domain. FliI contains a Walker A and B domain and mediates protein interactions by its N-terminus. **B**: Cpn0859 appears to dimerize, and interacts with the cytosolic domain of FlhA and the N-terminal 150 amino acids of FliI.

In *C. pneumoniae, Cpn0859 *is encoded directly downstream of the ATPase, which led us to explore any interactions Cpn0859 may have with other flagellar proteins. We initially found that Cpn0859 was able to interact with itself using a GST pull-down assay, suggesting that it could form dimers in solution. To confirm this observation we utilized formaldehyde fixation followed by PAGE analysis to visualize the formation of dimers. Using this method we saw that Cpn0859 migrated in two molecular forms, with sizes corresponding to both monomers and dimers. We then explored possible interactions between Cpn0859 and the other flagellar proteins and detected interactions of Cpn0859 with both FliI and FlhA, but not FliF. Cpn0859 bound to the N-terminal 150 amino acids of FliI and the cytoplasmic region of FlhA. The interaction of Cpn0859 with the cytoplasmic domain of FlhA was expected as FlhA is known to interact with soluble components of other flagellar systems [34]. We considered the possibility that Cpn0859 may in fact be the FliH ortholog in *C. pneumoniae *as Cpn0859 has minor sequence orthology to other FliH proteins, but after further investigation we found that Cpn0859 did not appear to play a regulatory role with FliI (data not shown). Figure 6 summarizes the interactions between FliI, FlhA and FliF (Figure 6A) and interactions between Cpn0859, FlhA and FliI (Figure 6B).

Bacterial type III secretion (T3S) and flagellar secretion systems are structurally similar, and may have a common ancestry [21]. Although *C. pneumoniae *does not contain a full repertoire of flagellar genes, it does encode a complete T3S system which most likely consists of specific protein complexes located in the inner membrane [16,20,23]. We have characterized an interaction of FliI with CdsL, the T3S ATPase tethering protein. The *C. pneumoniae *FliH ortholog has not yet been identified, and in the absence of FliH, CdsL may play a regulatory role for both FliI and CdsN. FliI also interacts with the CopN, the T3S plug protein, suggesting that FliI may be involved in either the secretion of effector proteins or regulation of the T3S system. YscU orthologs have a flagellar paralog, FlhB, and Cpn0322 is believed to be the *C. pneumoniae *YscU ortholog (CdsU). FlhB is known to interact with FlhA, but in *C. pneumoniae *no FlhB ortholog has been annotated. We found that FlhA interacts with CdsU, suggesting integration of FlhA into the inner membrane, associating with T3S components.

## Conclusions

Based on all of our observations, it is possible that the flagellar proteins may interact with components of the T3S injectisome, forming a hybrid structure, thereby playing an ancillary or accessory role in secretion of type III effectors across either the cytoplasmic or inclusion membrane. Another possibility is that there are other, as yet unannotated proteins that play a role in a putative flagellar system in *C. pneumoniae*. For example, along with the FliH/FliI complex that is formed in other bacteria, another protein, FliJ, which is a general chaperone, is believed to be involved in this complex [39,44]. FliJ has not been identified in *C. pneumoniae*. In the absence of a genetic manipulation system for the *chlamydiae*, direct evidence for the role of these flagellar proteins remains elusive. The fact that FliI is enzymatically active and forms complexes *in vitro *with other flagellar proteins, all of which are present in all other chlamydiae *sps*. studied to date, suggests that these proteins play an important role in chlamydial replication or survival. Further studies using heterologous systems and genetic complementation could help to decipher the exact role of these flagellar proteins in chlamydiae.

## Methods

### Expression Plasmids

*C. pneumoniae *CWL029 (VR1310:ATCC) (GenBank accession # AE001363) was the strain used to isolate genomic DNA for cloning and protein expression. Full length fliI, *Cpn0859, cdsL, copN, Cpn0322*, and fragments of *flhA*, *fliF*, and *fliI *were amplified from CWL029 using AttB-containing primers (Gateway; Invitrogen). The amplified products were cloned into pDONR_201 _(Gateway; Invitrogen) to generate pENT vectors. The pENT vectors were then used in LR reactions (Gateway; Invitrogen) to produce pEX vectors containing the genes of interest. We used either pEX_17 _(N terminal His tag) or pEX_15 _(N terminal GST tag) vectors for our protein expression. All constructs were confirmed by sequencing at the Molecular Biology Facility at McMaster University.

To identify protein interactions we utilized the bacterial-2-hybrid system [39]. Genes of interest were cloned into either pT18 or pT25 plasmids, each of which expresses a different fragment of adenylate cyclase. When these two plasmids are co-transformed, expressing the protein of interest fused to the adenylate cyclase fragment, any interaction between the two proteins results in production of cAMP. Increases in cAMP results in an increase in the β-galactosidase gene that can be monitored using β-galactosidase activity assays. pT18 and pT25 were digested with KpnI (New England Biolabs) as well as genes amplified from CWL029 (*fliI, flhA, fliF, cdsL, Cpn0322, copN*) that had a KpnI site designed into the primers. Ligation was performed overnight at 16°C using T4 Ligase (Invitrogen) and the resulting mixture was used to transform *E. coli *XL-1 cells and transformants were selected on 100 μg/μL ampicillin and 34 μg/μL chloramphenicol (Luria Bertani) LB plates. Plasmids were prepared using the GenElute Plasmid Miniprep Kit (Sigma).

### Protein Expression

All constructs were expressed in *E. coli *Rosetta pLysS. Expression plasmids were used to transform *E. coli *Rosetta pLysS and plated on LB plates containing 100 μg/mL ampicillin. LB broth (750 mL), containing antibiotics, was then inoculated with 12 mL of an overnight culture and grown at 37°C until they reached an optical density (OD)_600 _of approximately 0.8. Cultures were then cooled on ice to 20°C and induced with 0.2 mM of isopropyl β-D galactosidase (IPTG). Cultures were then incubated at 23°C for 2 hours and bacteria were harvested by centrifugation at 6500 × g for 10 minutes in a Sorvall RC-5B centrifuge and washed with ice-cold phosphate buffered saline (PBS). Bacteria containing His-tagged protein were resuspended in Binding Buffer (50 mM potassium phosphate pH 7.2, 150 mM KCl, 1 mM MgCl_2_) while the bacteria containing GST-tagged protein were resuspended in PBS and stored at -20°C until further use.

### Purification of Recombinant Proteins

*E. coli *pellets containing over-expressed proteins were thawed on ice and sonicated using a Fischer Scientific Sonic Dismembrator Model 100, followed by centrifugation at 20,000 × g for 40 minutes to remove insoluble material. Supernatants containing His-tagged protein were stored at 4°C for use in GST pull-down assays while the GST-tagged protein supernatents were filtered through 0.45 μm acrodisc filters (Pall Corporation) and incubated overnight at 4°C with 300 μL of Glutathione-agarose beads (Sigma). For GST pull-down assays, beads were blocked overnight in Tris Buffered Saline with 0.1% Tween-20 and 4% BSA and stored at 4°C until use. For ATPase activity measurements, glutathione beads were washed on a column with PBS + 0.1% Tween until the flow-through had an OD_280 _of less than 0.005. GST-tagged protein was then eluted off the beads using 1.5 μg/μL reduced glutathione (Sigma) and dialyzed against activity buffer (50 mM Tris-HCL pH 7.0, 5 mM MgCl_2_, 10 mM KCl). Purity was confirmed using SDS-PAGE and Coomassie blue staining.

### Dimerization Assay

In order to determine whether Cpn0859 formed dimers, formaldehyde fixation and non-denaturing PAGE were used. His-Cpn0859 was purified from Ni-NTA beads, dialyzed against PBS and concentrated using Amicon 10 kDa (Millipore) concentrators to a final concentration of 1 μg/μl. Formaldehyde was added to purified His-Cpn0859 to a final concentration of 10% and fixation was allowed to continue for 10 minutes. Samples containing 1 μg of Cpn0859 were electrophoresed on an 8% non-denaturing PAGE and visualized by Western blot using anti-His antibody (Sigma).

### ATPase Activity

ATP hydrolysis by GST-FliI purified from glutathione-agarose beads was measured using a malachite green assay (R & D Systems). For all experiments, the specific activity was determined using the equation of a standard line generated using phosphate standard (R & D Systems). Reaction mixtures contained 150 ng of GST-FliI, 4 mM ATP, 50 mM Tris-HCL pH 7.0, 5 mM MgCl_2_, and 10 mM KCl. The reaction mixture (1 mL) was incubated at 37°C for 1 hour and 50 μL of the mixture was taken for inorganic phosphate determination at various time points. The reaction was stopped by the addition of 10 μL of Malachite Green Reagent A followed by 10 μL of Malachite Green Reagent B and incubated at room temperature for one minute before an OD_610 _reading was taken, according to the manufacturer's instructions. For the negative control, purified FliI was digested for 10 minutes at 37°C using Proteinase K (Invitrogen). Also, as a negative control, another GST-tagged protein (CopN) known not to have ATPase activity was purified in the same manner and tested for activity. ATPase activity was expressed as μmol phosphate released min^-1 ^mg^-1 ^of protein, and all experiments were performed in triplicate.

### GST Pull-down Assays

To examine the interaction of the flagellar proteins, GST pull-down assays were performed as described previously with the following modifications [20]. Briefly, glutathione agarose beads (30 μL) bound to fifty nanograms of GST tagged FliI, Cpn0859, or FlhA was used in the assay. The beads were incubated overnight at 4°C with the *E. coli *lysate expressing the His-tagged proteins. The beads were collected by centrifugation and washed with increasing concentrations of NaCl to eliminate spurious protein interactions. All proteins were eluted from the Glutathione beads and electrophoresed on an 11% SDS-PAGE gel before being probed for His-tagged protein. As a negative control, GST alone was incubated on beads with the *E. coli *lysates.

### Bacterial-2-Hybrid Assay

The bacterial-2-hybrid assay uses protein-protein interactions to bring two fragments of adenylate cyclase catalytic domain together to produce cAMP, stimulating β-galactosidase activity. β-galactosidase activity is therefore a representation of protein interaction. This protocol was performed as described by Karimova et al, 2005 [45]. Briefly, *E. coli *DHP-1 cells (an adenylate cyclase deficient cell line) were transformed using pT18-FliI/pT18-FlhA/pT18-FliF and either pT25-FlhA or pT25-FliF and selected with 100 μg/μL ampicillin and 34 μg/μL chloramphenicol. Three individual colonies were selected from each plate and grown overnight in 3.0 mL of LB at 30°C in the presence of 0.5 mM IPTG plus appropriate antibiotics. Overnight culture (200 μL) was diluted 1 in 5 into M63 buffer (75 mM (NH_4_)_2_SO_4_, 110 mM KH_2_PO_4_, 200 mM K_2_HPO_4_, 5 mM FeSO_4_-7H_2_O) and the optical density at 600 nm was recorded. The cells were permeabilized using 0.01% Toluene and 0.01% SDS. For the reaction, 50 μL of the permeabilized cells were diluted into 450 μL of LB broth. The diluted cells were then added to 500 μL of PM2 (70 mM Na_2_HPO_4_-H2O, 30 mM NaHPO_4_-H2O, 1 mM MgSO_4_, and 0.2 mM MnSO_4_) buffer containing 100 mM β-mercaptoethanol. The reaction was initiated by adding 250 μL of 12 mg/mL *ortho*-nitrophenyl-β-galactoside and allowed to continue for 15 seconds at 28°C. The reaction was stopped by the addition of 500 μL of 1.0 M Na_2_CO_3_. The absorbance was measured at 420 nm and the β-galactosidase activity was expressed as units of β-galactosidase activity per milligram of bacteria. Empty pT18 and pT25 vectors were transformed into *E. coli *DHP1 cells as a negative control and pT18-CopN and pT25-CdsN were used as a positive control (38). The cutoff for a positive interaction (677 units activity/mg bacteria) was determined as the mean plus two standard deviations of the negative control values obtained from 20 assays.

## Authors' contributions

CS performed most of the experimental work. DB aided in the bacterial-2-hybrid studies. JG cloned and expressed Cpn0322. RT explored potential oligomerization of FliI. JM coordinated the work and edited the manuscript. All authors read and approved of the final manuscript.
